# In Vitro Assessment of Bioactive Glass Coatings on Alumina/Zirconia Composite Implants for Potential Use in Prosthetic Applications

**DOI:** 10.3390/ijms20030722

**Published:** 2019-02-08

**Authors:** Francesco Baino, Joaquim Minguella-Canela, Feza Korkusuz, Petek Korkusuz, Berna Kankılıç, María Ángeles Montealegre, M. Antonia De los Santos-López, Chiara Vitale-Brovarone

**Affiliations:** 1 Politecnico di Torino, Department of Applied Science and Technology, Torino, Corso Duca degli Abruzzi 24, 10129 Torino, Italy; francesco.baino@polito.it (F.B.); chiara.vitale@polito.it (C.V.-B.); 2 Centre CIM/Departament d’Enginyeria Mecànica, Universitat Politècnica de Catalunya, Av. Diagonal, 647, 08028 Barcelona, Spain; tania.santos@upc.edu; 3 Department of Sports Medicine, Medical Faculty, Hacettepe University, Sihhiye, Ankara 06100, Turkey; feza.korkusuz@gmail.com; 4 Department of Histology and Embryology, Medical Faculty, Hacettepe University, Sihhiye, Ankara 06100, Turkey; petek@hacettepe.edu.tr; 5 Department of Biotechnology, Graduate School of Natural and Applied Sciences, Middle East Technical University, Cankaya, Ankara 06800, Turkey; uysalberna@yahoo.com; 6 AIMEN Technology Centre, Relva 27A—Torneiros, 36410 Porriño, Spain; mamontealegre@ikergune.com

**Keywords:** Bioceramics, Bioactive glass, Scaffold, In vitro, Bioactivity, Prosthesis, Coating

## Abstract

Achieving the stable osteointegration of prosthetic implants is one of the great challenges of modern orthopedic surgery. The fixation of ceramic acetabular cups of hip joint prostheses is usually achieved using a metal shell provided with screws or pegs that penetrate into the host pelvic bone. The deposition of bioactive coatings on the implant surface to be put in contact with bone could be a valuable strategy to promote a more “physiological” osteointegration. In this work, bioactive glass porous coatings were manufactured on the top of alumina/zirconia composite implants by two different methods, i.e., sponge replication and laser cladding. The coated samples underwent immersion studies in Kokubo’s simulated body fluid (SBF) to assess in vitro bioactivity and were found to exhibit an excellent hydroxyapatite-forming ability, which is key to allow bonding to bone. Biological tests using mesenchymal stem and osteoblast-like cells revealed the good biocompatibility of both types of materials. Furthermore, a higher level of mineralization was induced by the sponge-replicated coatings at 10 days. Overall, these results are highly promising and encourage further research on these materials.

## 1. Introduction

Prosthetic implants are of great importance nowadays due to the increase of the elderly population worldwide with associated age-related musculoskeletal pathologies. In all of their forms and applications, implantable materials can be used for many purposes, from healing assistance to the complete replacement of human bones and joints in order to enhance the quality of living and healthy ageing [1].

The mechanical properties of the different materials used in bone prostheses must be sufficient to comply with the structural conditions required once the prostheses are implanted in a person. Ideally, the mechanical properties of the implant should be as similar as possible to those of host bone. The mechanical properties of bones depend on their density, which may significantly vary in the skeleton, according to the main function they have to accomplish. There are estimations in the literature of the Young’s modulus and strength for different kinds and parts of bones. The cortical bones have a Young’s modulus in the range of 1–20 GPa and a strength in the range of 1–100 MPa [2]. The cancellous bones, being significantly more porous (up to 80% in vertebral bodies), have a Young’s modulus in the range of 0.1–1.0 GPa and a maximum yield strength in the range of 1–10 MPa [3].

Metals, ceramics, and polymers can all be used to manufacture bone implants by different production methods, which impose several constraints on the performance of the parts achieved [4].

Metal components are used in more than 70% of prosthetic implants [5]. Nevertheless, metallic materials and their alloys have a high Young’s modulus and high maximum yield strength compared to bone. The Young’s modulus of titanium, for example, is within 100–120 GPa [6], which causes a mismatch with the stiffness of bone. Furthermore, metals can be susceptible to corrosion and wear in vivo and cannot bond to living bone; these limitations can be mitigated by depositing coating materials that have a protective effect and exhibit a more similar chemical composition and fine microstructure to the bones, thereby promoting osteointegration. From a technological viewpoint, depositing a ceramic layer on the surface of metallic implants is challenging due to the significant mismatch between the thermal expansion coefficients of the materials involved, which leads to difficult adhesion [7]. Good results were reported for plasma-sprayed hydroxyapatite coatings on metallic joint prostheses in terms of bone-implant fixation, but there still are some concerns on the stability of these coatings in the long term [8].

Polymers exhibit an excellent biocompatibility with cells and tissues, but typically suffer from poor structural behavior. In the last years, polymers have been extensively investigated for bone-tissue applications. Natural polymers are reported to have problems of structural instability due to progressive resorption over time once implanted in vivo [9]. Synthetic polymers can overcome this drawback and their properties can be finely tailored by acting on the composition and synthesis process [10,11]. The Young’s modulus of biomedical polymers is typically in the range of 0.35 to 7 GPa [12], which better fits that of bone as compared to metals, but the strength often still remains inadequate for load-bearing applications.

Ceramic-based prostheses open a wide opportunity window for achieving a better biomimetic behavior than any other solution. Ceramic materials such as calcium phosphates, bioactive glasses, alumina, and zirconia have been studied for medical applications for five decades [13]. Polymer–ceramic composites are further possible combinations for achieving biomedical materials with finely tuned bioactive and mechanical properties [14]; however, these composites are only used for non-load-bearing applications [15].

General methods for manufacturing ceramic-based prostheses include molding, additive manufacturing, machining, or a combination of two or more of these processes, with heat treatments in the form of oven sintering [16,17,18,19].

Alumina, zirconia, and composites thereof have been found to be highly suitable for fabricating femur heads and ceramic inserts (acetabular cups) in hip joint prostheses due to their anti-friction properties, high strength, and excellent biocompatibility in the body [20]. At present, ceramic acetabular cups are fixed to the patient’s pelvic bone intraoperatively by using a metal shell, called “metal-back”. This procedure can carry the risk of incorrect positioning of the implant (miscentering) and mechanical damage to the host bone. The need for the metal-back and relevant disadvantages could be overcome by the deposition of a bioactive coating on the outer surface of the acetabular cup for promoting osteointegration [21].

**Figure 1:**
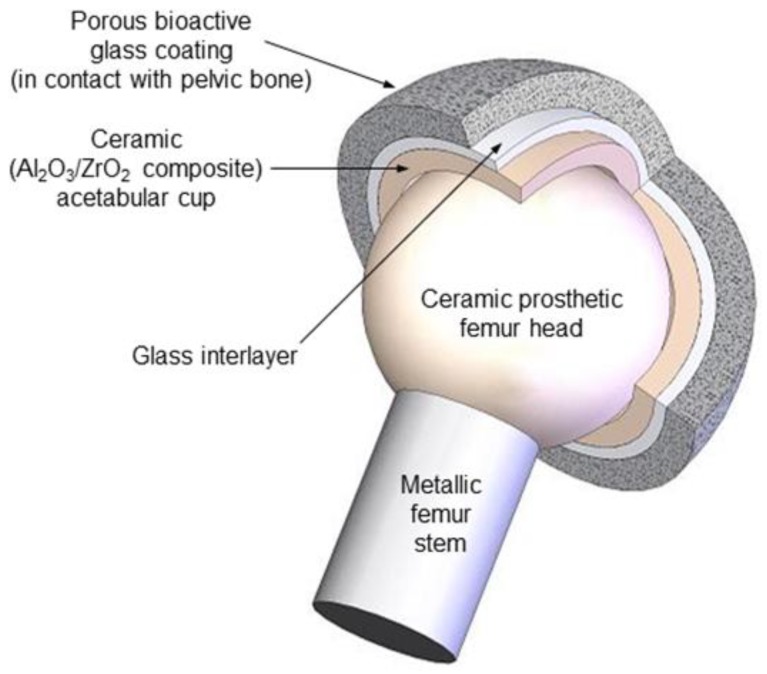
Single-piece multi-layered acetabular component of the innovative hip joint prosthesis proposed by the authors: the ceramic cup is coated by a bone-like porous layer of bioactive glass that is potentially able to bond to the host bone.

This work is the third part of a trilogy focused on demonstrating the feasibility of the above-mentioned approach, in which bioactive glasses are used to innovatively manufacture multi-layered coatings mimicking the architecture of cancellous bone [22,23]. A scheme of this concept is illustrated in Figure 1. A couple of previous studies reported the successful fabrication of trabecular coatings on ceramic acetabular cups by sponge replication [14] and laser cladding [24]. The present work moves a step forward and aims at assessing the in vitro bioactivity (i.e., the HA-forming capability in simulated body fluid) and biological compatibility of both types of coatings in a simplified flat configuration.

## 2. Results and Discussion

**Figure 2:**
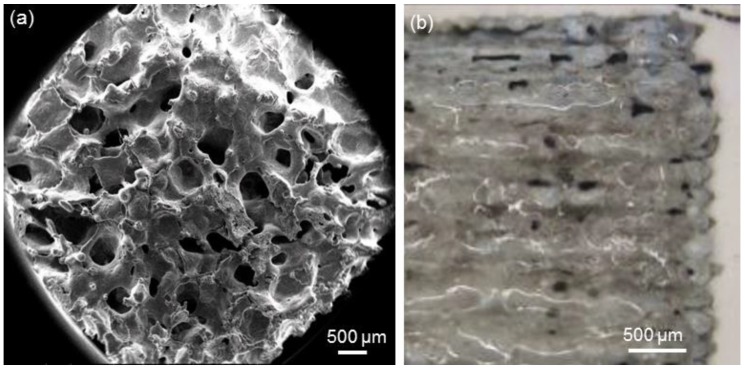
Morphologies of the top porous coatings produced by (**a**) sponge replication and (**b**) laser cladding on the alumina/zirconia composite pellets.

The methods adopted for manufacturing the top porous layers on the alumina/zirconia composite pellets, i.e., sponge replication and laser cladding, led to significantly different results, as illustrated in Figure 2. The sponge replica method allows a 3D porous structure with large and open macropores (100–500 µm) to be obtained, which closely mimics the trabecular architecture of cancellous bone (Figure 2a). On the contrary, the surface pores of laser-cladded coatings are formed at the intersections of the fused glass tracks and as a result of PVA burn-off (Figure 2b). These morphological results, which are referring to flat coatings, are consistent with previous observations on a curved geometry (acetabular cup) [16,17,18,19,20,21,22,23,24].

The final scope of the multi-layered prosthetic devices presented in this work is to stimulate bone in-growth and regeneration, thus potentially allowing the direct attachment of the implant to the host bone. As mentioned in the introduction, on the basis of robust experimental work carried out by many research groups over the last three decades [25,26], it is assumed that the formation of a hydroxyapatite (HA)-like layer on the surface of biomaterials in SBF is reliable proof of the capability to bond to living bone in vivo. Therefore, the assessment of in vitro bioactivity of the glass coatings produced in this work is of the utmost importance to support their medical suitability.

Following contact with a physiological fluid, the in vitro formation of HA on the surface of bioactive glasses occurs through a sequence of five consecutive stages [27]:

1. Na^+^ cations are rapidly released and replaced by H+ ions from the solution;
2. the corresponding increase of local pH promotes the breaking of Si–O–Si bonds on the glass surface and the release of soluble silica;
3. some of the surface Si–OH groups formed in steps (1) and (2) condense to form a hydrated silica-rich layer on the glass surface, which is depleted of modifiers ions;
4. calcium and phosphate ions are released through the silica gel layer, which incorporates other Ca^2+^ and (PO_4_)^3−^ ions from the solution to form an amorphous calcium phosphate phase;
5. this amorphous film may incorporate additional carbonate ions from the solution and eventually crystallize into hydroxycarbonate apatite.

Silicate glasses are characterized by a continuous network, fully interconnected in 3D, with every silica tetrahedron linked by bridging oxygens (BOs) to four adjacent tetrahedra. The addition of alkali or alkaline-earth metal cations (the so-called “modifiers”) breaks the silicate network by replacing Si–BO–Si bonds with Si–NBO, where NBO is a non-bridging oxygen. Ionic bonds between NBOs and modifiers ensure the local charge balance and the overall charge neutrality. Although being weaker than the covalent Si–O bonds, the ionic interaction between NBOs and modifiers is extremely important as far as glass reactivity is concerned [28]. The larger the number of NBOs in the glass structure, the higher the solubility of small silica fragments in SBF; hence, a high degree of glass network fragmentation is strongly correlated to the high bioactivity of the glass.

**Figure 3:**
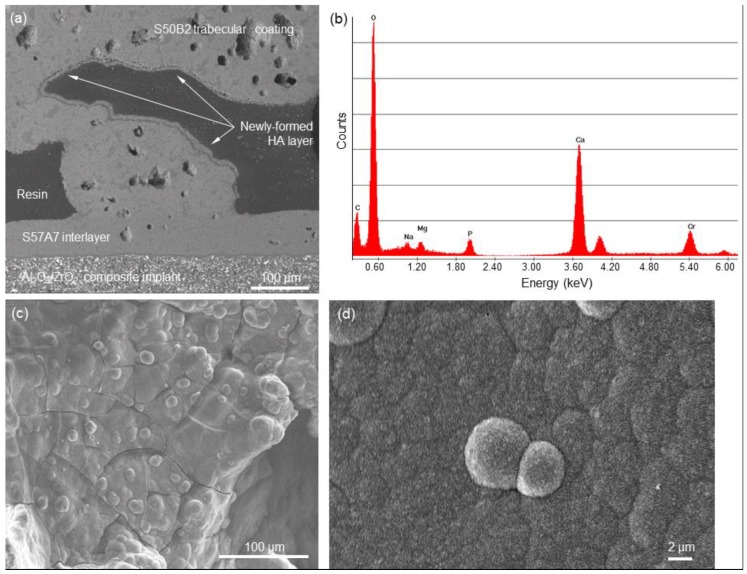
In vitro bioactivity test: (**a**) SEM cross-sectional image of a multi-layered sponge-replicated sample after being soaked for seven days in SBF and (**b**) compositional analysis (EDS) of the newly-formed HA layer (the peak of Cr is due to ultrathin metal coating needed for the analysis); (**c**) and (**d**) SEM micrographs of the newly-formed phase on the top of porous S50B2 glass coating at different magnifications (800 and 10,000×).

The results of in vitro bioactivity studies in SBF are shown in Figure 3. The SEM back-scattering mode (Figure 3a) emphasized the presence of a newly-formed phase on the trabeculae of the S50B2 porous coating. Compositional analysis was performed by Energy Dispersive Spectroscopy (EDS). EDS revealed that this new phase is rich in calcium and phosphorus, with a Ca-to-P atomic ratio of 1.2. This value is lower than the Ca-to-P ratio of stoichiometric HA (1.67) and indicates that, actually, a Ca-deficient HA is formed on this material. This situation is quite common for the HA-like phase formed on the surface of bioactive glasses, as observed in previous investigations on other silicate glass compositions [29,30]. The surface morphology of this new phase, shown in Figure 3, exhibits the typical “cauliflower” appearance of bone-like HA, formed by globular agglomerates in which needle-like nanocrystals can be detected (Figure 3d).

The presence of an HA-like layer on the struts of the porous coating is expected to play a key role in promoting colonization by bone cells in vivo, as it was demonstrated that osteoblasts attach and spread preferably on nanocrystalline HA due to its chemical and crystallographic similarity to bone mineral [31]. In this regard, the HA layer can be interpreted as a “biomimetic skin” that continuously and homogeneously covers all the surfaces of the porous coating (see Figure 3c). It is noteworthy that the peak of silicon, which is indicative of the glass and/or silica gel layer, is no more visible in the EDS plot reported in Figure 3b.

Figure 3a also reveals the excellent joining between the three layers (ceramic substrate + interlayer + porous coating) that constitute the device. This confirms that the integrity of the implant is retained after immersion is biological fluids, which is of crucial importance for the safety of any clinical application.

The mechanical suitability of these materials for potential use in prosthetic and bone implant applications was evaluated in previous studies [16,32]. Tensile (pull-out) tests allowed the authors to determine that the adhesion strength of S57A7 coatings on alumina was about 27 MPa [16]. Interestingly, this value is two times higher than that recommended by the ISO13779 standard for HA coatings on surgical implants [33]. Therefore, the high bonding strength of S57A7 coatings supports their mechanical suitability for the intended biomedical application.

The tensile strength of porous S50B2 samples was assessed to be in the range of 5.4 to 7.8 MPa. This value is comparable to the tensile strength of bovine spongy bone (7.6 ± 2.2 MPa [34]) and human trabecular bone (10–20 MPa [35]).

**Figure 4:**
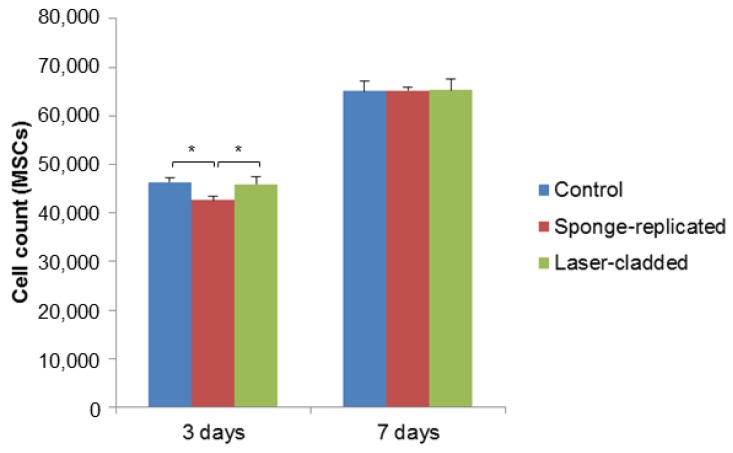
MSC proliferation test (cell count) on S50B2-coated samples (*p* < 0.05).

**Figure 5:**
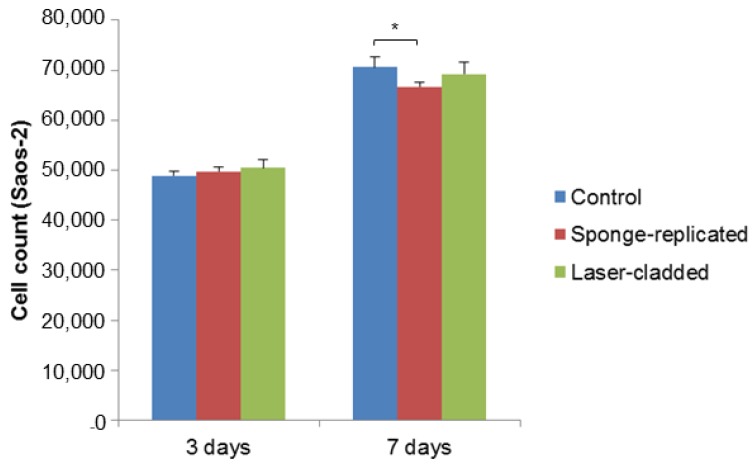
Saos-2 cells proliferation test (cell count) on S50B2-coated samples (*p* < 0.05).

The biological compatibility of the multi-layered implants produced by sponge replication and laser cladding was assessed by early cell seeding experiments. As shown in Figure 4; Figure 5, both cell types attached and proliferated over time on all substrates, with moderate or no differences in terms of cell count. The number of MSCs grown on the sponge-replicated coating at three days is lower than that assessed on the laser-cladded implant. On the contrary, no statistically significant differences were found at the same time point if the samples were seeded with Saos-2 cells. No statistically significant differences were found at seven days for MSCs seeded on both sample types and the control; a slight decrease of cell number was instead observed for Saos-2 cells seeded on the sponge-replicated coating compared to the control.

As a limitation to this study, cell adhesion tests were conducted in 12-well plates. Each well had a surface area of 3.8 cm^2^, with a 22 mm diameter. In the control group, cells were seeded into the well without any material and thus had a broader surface to grow. On the contrary, for the experimental groups, cells were seeded directly on top of the tested material that was placed in the center of the well. Since the tested materials only had a 8.6 mm diameter, the surface area of tested material was lower than that of the well. MSCs and Saos-2 cells proliferated on both material types, but the proliferation rates were not statistically significantly higher than the control group due to the presence of the material itself. Another limiting factor could be related to the release of alkaline ions from the materials during the proliferation study. This may have changed the pH of the environment towards alkalinity and, thus, could have caused slight differences in cell proliferation rates.

**Figure 6:**
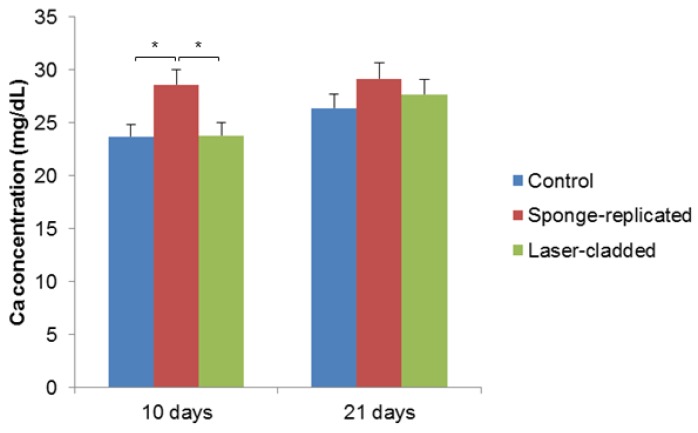
Mineralization test on S50B2-coated samples carried out by using MSCs (*p* < 0.05).

Mineralization tests, reported in Figure 6, suggest that the different coating morphologies (foam-like vs. laser-cladded) could play a role in promoting the calcium nodule formation by MSCs, which is a first index of cell differentiation towards osteoblastic lineage. It can be observed that the mineralization ability of the cells grown on sponge-replicated coatings at 10 days is higher compared to laser-cladded samples. This effect can be explained considering that the foam-derived coatings are more porous and have a higher specific surface area available for ion-exchange phenomena compared to the laser-cladded type (see the different morphologies reported in Figure 2). In this regard, the ionic species released from bioactive glasses were demonstrated to stimulate cell differentiation, as well as the genes of cells towards paths of osteogenesis and self-repair [36]. Ion leaching tests could be carried out in future studies to further elucidate these aspects.

**Figure 7:**
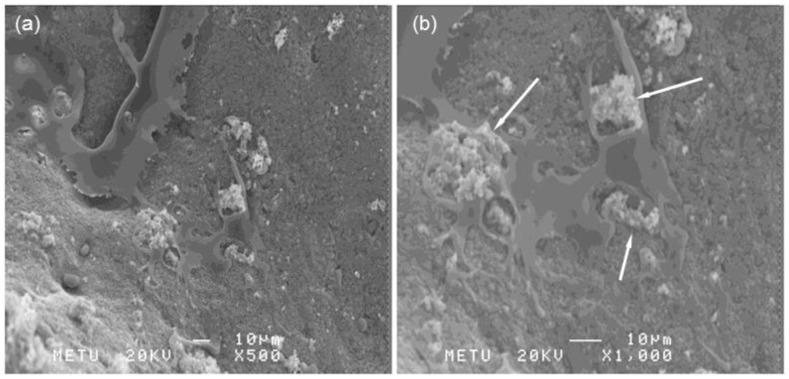
SEM images of MSCs after 10 days of incubation on S50B2 scaffolds: (**a**) magnification at 500× and (**b**) magnification at 1000×. The white arrows in (**b**) indicate calcium nodules.

Figure 7 shows that MSCs appear closely attached to the surface of the S50B2 coating. Cells exhibit a flattened morphology, with a lot of filopodia and lamellipodia. The flat and extended cell morphology confirms the viability of MSCs seeded on the coatings. The presence of calcium nodule agglomerates can also be seen, in accordance with the mineralization test results.

## 3. Materials and Methods

### 3.1. Preparation of the Alumina/Zirconia Composite Substrates

The raw material chosen for producing the ceramic substrates was a composite powder consisting of alumina and zirconia in a 75:25 (wt%) ratio. The raw powder aggregate was compressed in a hydrostatic press brake for obtaining cylindrical preforms, which exhibited a very fragile and brittle behavior. Therefore, a pre-sintering process was undertaken in order to let the samples achieve the necessary hardness and cohesion properties to be machined. The pre-sintering process consisted of heat treatment in an oven with forced air circulation with two temperature-targeted steps. The first step was a stop at 100 °C for 2 h to evaporate the humidity traces that could have been residing in the cylinders. The second step was a stop at 1200 °C for 2 h, allowing minimum consolidation of the geometry. After that, the cylinders were stabilized and allowed to cool down to room temperature, thus becoming the “green” ceramic cylinders. The total time of the heat curve was 17 h, requiring 9 h to meet the second-step target temperature with a low heating rate and the remaining 6 h to cool down.

**Figure 8:**
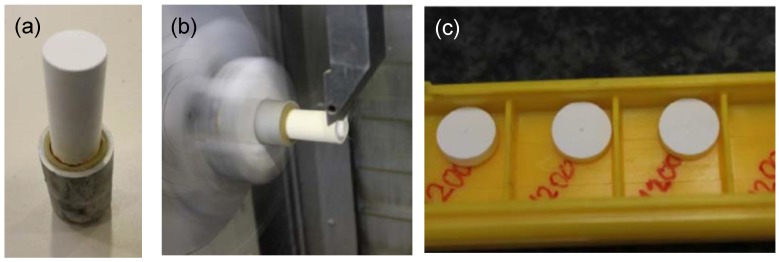
Preparation of the composite alumina/zirconia pellets: (**a**) Green cylinder subjected to a turning tooling; (**b**) turning operation for obtaining the pellets; (**c**) disk-shaped samples obtained.

Then, the green ceramic cylinders were subjected to special turning tooling, developed on purpose for this application and processed in a multitasking center (Mori Seiki NT3150 DCG, 7-axis) [37]. The turning operations allowed several tens of pellet to be achieved with the desired geometry (small disks, see Figure 8).

Following this, the main sintering process was undertaken. It consisted of heat treatment in an oven with forced air circulation with a single stop at 1600 °C for 2 h. The total time of the heat curve was 19 h, requiring 11 h to meet the target temperature with low-slope heating and the remaining 6 h to cool down. During the preliminary sintering tests, a contraction factor of about 30% was observed in the external dimensions of the pellets. This phenomenon has to be taken into account to include a surplus factor in the turning operation so as to correctly achieve the nominal diameter. The samples used had dimensions of a 8.6 mm diameter and a 3 mm thickness.

### 3.2. Preparation of the Glasses

Two glass formulations (Table 1) were selected to produce multi-layered coatings on the alumina/zirconia composite substrates. The rationale behind the design of these glass compositions and the relevant characterization results are described elsewhere [32]. Both glasses were produced by using a standard melting method in a platinum crucible. The raw precursors (high-purity powders purchased from Sigma-Aldrich, St. Louis, MO, USA; see Table 1) were homogeneously mixed in the crucible and melted at 1450 °C for 1 h in air. The melt was then quenched in distilled water to produce a frit that was ball milled (Pulverisette 6, Frtisch, Germany) and sieved either below 32 μm or within 60–150 μm by stainless steel sieves (Giuliani Technologies Srl, Torino, Italy) for further processing.

### 3.3. Fabrication of the Coatings

Two kinds of manufacturing processes were used to fabricate the glass coatings, i.e., (i) a proper combination of enameling and sponge replication, or (ii) laser cladding.

#### 3.3.1. Method 1: Enameling and Sponge Replication

**Figure 9:**
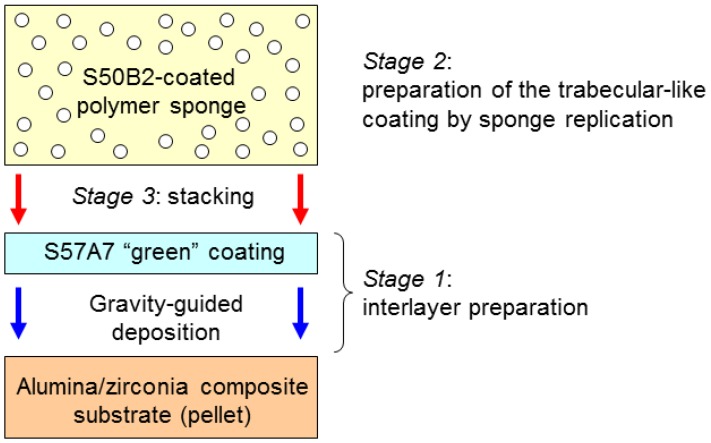
Fabrication processes of the coatings via Method 1: enameling + sponge replication. Stages 1, 2, and 3.

The fabrication method based on enameling and sponge replication can be divided into four stages; the first three stages are also illustrated in Figure 9.

• Stage 1

S57A7 “green” dense coating (interlayer) was prepared on the alumina/zirconia composite pellet by gravity-guided deposition of glass particles (<32 μm) suspended in ultrapure ethanol via sonication. After ethanol removal, the green coating was thermally-treated at 1000 °C for 1 h (heating rate 5 °C/min) to allow their consolidation (sintering of glass particles and adhesion of the coating to the substrate). The presence of this (inter)layer is crucial to maximizing the adhesion between the ceramic substrate and the top porous coating, as described in [16].

• Stage 2

A commercial open-cell polyurethane sponge (apparent density 20 kg/m^−3^) was manually cut into 5-mm thick strips, from which small disks were obtained by using a metallic punch. These porous polyurethane blocks were dipped into a water-based slurry containing S50B2 particles (<32 μm) and polyvinyl alcohol (PVA) used as a binder (glass/PVA/water = 40:6:54 (wt%)). Each sponge disk was soaked in the glass suspension for 60 s, extracted from it, and then axially compressed (60% of the height) to squeeze the excess slurry out of the foam pores. Compression was performed three times, alternating the face put in contact with the piston in order to homogeneously remove the slurry. This sequence of actions was repeated three times, followed by a final cycle of dipping into the glass suspension without any compression. It is noteworthy that the alumina/zirconia ceramic substrates had a nominal diameter of 8.60 mm, but the sponge to stack on them (Stage 3 in Figure 9) had to be properly oversized (the punch diameter was 10.0 mm) for taking into account the volumetric shrinkage that occurs during sintering.

• Stage 3

The S50B2-impregnated sponge (prepared in the Stage 2) was stacked onto the S57A7 glass-coated pellet prepared in Stage 1.

• Stage 4

The three-layer system was thermally treated in an electrically-heated furnace at 1100 °C for 1 h (heating rate 5 °C/min) in air to allow the burning-out of the polymeric sponge and the sintering of the glass powders. As a result, the three layers were joined together and a positive glass-derived replica of the porous polymeric template was obtained on the top.

#### 3.3.2. Method 2: Laser Cladding

**Figure 10:**
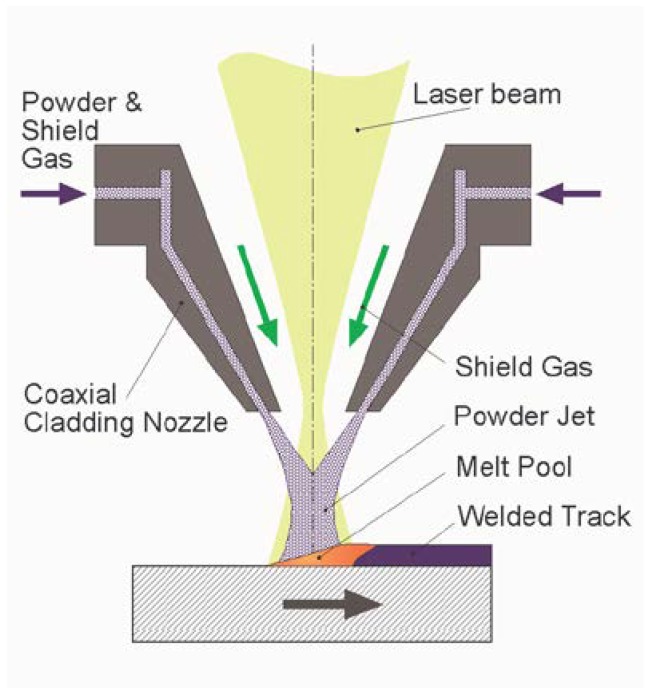
Fabrication processes of the coatings via Method 2: laser cladding.

Manufacturing of dense S57A7 glass coatings (interlayer) by laser cladding involves spraying of the glass powder over the bioceramic surface and the simultaneous action of a laser beam that melts the particles together (Figure 10). Before reaching the target substrate, glass particles are ejected through a narrow nozzle and, thus, there is the risk of nozzle blocking as they tend to spontaneously agglomerate. In order to avoid that, the particles were kept in an oven at 100 °C for 6 h to remove humidity prior to being used. Additionally, the powder feeder was kept at 90 °C during the entire processing time in order to avoid moisture-related problems.

The laser source was an ROFIN CO_2_ laser DC035 operating in the infrared region (wavelength 10.6 µm). In the present work, the pulse frequency was set at a target of 5 kHz, with a maximum power of 350 W (thus, the duty cycle varied from 20 to 35%). In order to avoid directional heterogeneity during the sample’s processing, the laser beam polarization was transformed from linear to circular by means of a Cu mirror.

The powder feeder utilized (Medicoat) can introduce particles into the interaction zone by the means of a stream of a protective inert gas (argon). For the samples required, the feeder was filled with the S57A7 powder sieved within 90–150 µm. The powder nozzle utilized (coaxial nozzle COAX 8, Fraunhofer IWS) worked attached to the laser processing optics. The laser beam was focused to a spot of 200 µm by a parabolic Cu mirror. The glass particles were sprayed during the operation of the system in a coaxial direction to the laser beam. In this way, both the beam and glass reached the substrate at the same incidence point. During the samples’ preparation, the values chosen for the working parameters ranged between 2 and 3 g/min for the glass mass flow and 3 L/min for the argon volumetric carrier flow from the feeder to the nozzle and 10 L/min for the argon volumetric flow for the shielding gas during the feeding.

The manipulation of the operation head—including both the laser optics and the powder nozzle set- was performed in an integrated manner with the handling of the working platform—ceramic substrate to be coated- by means of a five-axis CNC machine architecture (Lantec). This configuration made it possible to generate laser-cladded glass tracks on any of the visible surfaces of the ceramic pellets, and so to obtain dense coatings by overlapping several of those tracks. Laser cladding was also experimented with to fabricate porous glass coatings over the previously-deposited dense interlayer. The experimental setup was similar to that used to prepare the dense coating, except for the glass powder spraying, which was replaced by the manual deposition of a high-solid-load slurry with 4 wt% of PVA as a binder. The porosity was then created as a result of inter-particle voids left behind by water evaporation (during post-deposition drying) and PVA burn-off due to laser scanning. The procedure adopted here was analogous to that applied to curve surfaces in a previous work [24].

### 3.4. In Vitro Bioactivity

The samples were soaked in simulated body fluid (SBF) for seven days to assess their apatite-forming ability (i.e., bioactivity). Static experiments were carried out by immersing each specimen in 30 mL of SBF prepared according to the Kikubo’s protocol [26] and maintained at 37 °C (body temperature). The solution was refreshed twice a week to simulate fluid circulation as it occurs in the human body. At the end of the experiment, the samples were gently rinsed with distilled water and left to dry at room temperature, before being studied by scanning electron microscopy (SEM; Supra^TM^ 40, Zeiss, Oberkochen, Germany) incorporating Energy Dispersive Spectroscopy (EDS). The sample surface was sputter-coated with chromium prior to the SEM-EDS analysis.

Before being assessed by means of SEM-EDS, several samples were prepared in a matrix of epoxy resin (Struers Epofix), cut by a diamond wheel (Struers Accutom 5) and polished using SiC grit papers #600 to #4000 (PACE Technologies, Tucson, AZ, USA).

### 3.5. Biological Tests

Both types of coatings underwent in vitro biological assessment to evaluate cell adhesion, proliferation, and mineralization ability.

#### 3.5.1. Cell Isolation, Expansion, and Culture

Bone Marrow (BM) Mononuclear Cells (MNC) (Lonza Group, Basel, C.H.) were isolated by density centrifugation with Ficoll hypaque (1.077 g/L; Biochrom AG, Berlin, Germany). Cell culture medium consisted of low-glucose Dulbecco’s modified Eagle medium (LG-DMEM; Biochrom AG, Berlin, Germany), along with 10% fetal calf serum (FCS; Biochrom AG, Berlin, Germany) and 1% penicillin/streptomycin (Biochrom AG, Berlin, Germany). The complete medium was replaced every three to four days. Cells were harvested with 0.25% trypsin/1 mm EDTA and re-plated when adherent cells reached sub-confluence. From passage 3 (P3), mesenchymal stem cells (MSCs) were characterized according to the positive expression of CD 90, CD 105, CD 73, CD 44, and CD 49e surface markers with a FACSAria flow cytometer (Becton Dickinson, Franklin Lakes, N.J., USA). Primary human osteosarcoma cell line Saos-2 was purchased from Sigma-Aldrich (Sigma Aldrich, St. Louis, MO, USA).

#### 3.5.2. Adhesion Test

Total 308000 MSCs and 308000 SaOS-2 cells were used. Adhesion assays were carried out on days 3 and 7. Control groups were MSCs and Saos type 2 cells cultured in the appropriate medium without samples. Tests were carried out in triplicate for each material and cell group. The cells were cultured with MSC or SaOS-2 medium, according to cell type. MSC culture medium consisted of 52.8% Dulbecco’s Modified Eagle Medium (DMEM) with 1 g/L glucose (Lonza, Basel, Switzerland), 35.2% MCDB-201 medium (Sigma Aldrich, St. Louis, MO, USA), 10% heat inactivated fetal bovine serum (FBS) (Sigma Aldrich, St. Louis, MO, USA), 1% penicillin/streptomycin solution (Biochrom AG, Berlin, Germany), and 1% l-glutamine (Biochrom AG, Berlin, Germany), while SaOS-2 culture medium consisted of 89% DMEM with 4.5 g/L glucose (Sigma Aldrich), 10% heat inactivated FBS, and 1% penicillin/streptomycin solution. Every three to four days, the medium was replaced. The plates were incubated at 37 °C with relative humidity under an atmosphere of 5% CO_2_. On the third and seventh days, the wells were washed with PBS (Oxoid, Hampshire, United Kingdom) and 1 mL Trypsin/EDTA (Thermo Fisher, Waltham, MA, USA) was applied. A 50 µL cell suspension was mixed with 50 µL Trypan blue (Biological Industries, Beit HaEmek, Israel) in a centrifuge tube and the cells were counted on Thoma lamina.

#### 3.5.3. SEM Investigation

Then, 40,000 MSCs were cultured on the glass-coated ceramic pellets. The cells were incubated with samples and growth medium (DMF10) composed of 10% fetal bovine serum (FBS) (Invitrogen, Carlsbad, CA, USA), 1% penicillin-streptomycin (Biochrom AG, Berlin, Germany), and DMEM-LG (Biological Industries, Beit HaEmek, Israel) containing 1% l-glutamine (Biochrom AG, Berlin, Germany) in six-well plates. On the third day, medium was removed from the wells and samples were washed with PBS two times. After that, 3 mL 2.5% glutaraldehyde (Merck, Darmstadt, Germany) in distilled water was added to the wells and left for 30 min. Then, the cells were again washed with PBS and thereafter were dehydrated with graded ethanol series in distilled water. The samples were incubated in 50% EtOH (5 min), 70% EtOH (5 min), 80% EtOH (10 min), 95% EtOH (10 min), and 100% EtOH (20 min). Air-dried samples were prepared for SEM analysis by sputter-coating with gold. The gold-coated samples were analyzed by a JEOL scanning electron microscope JSM 6400 (Jeol Ltd., Akishima, Japan).

#### 3.5.4. Mineralization Assays

In total, 375000 MSCs were used. Time points for the assay were day 10 and 21. The control group was blank MSCs incubated in the medium without samples. The cells were incubated with samples and growth medium in 12-well plates for the first day. Every three to four days, the medium was replaced. When the cells reached 50–60% confluence, differentiation medium containing DMEM-LG, 10% FBS, 100 nmol/L dexamethasone (Sigma-Aldrich, St. Louis, MO, USA), 10 mmol/L beta glycerophosphate (Sigma-Aldrich, St. Louis, MO, USA), and 0.2 mmol/L ascorbic acid (Sigma- Aldrich, St. Louis, MO, USA) was added to the wells. On the tenth and twenty-first days, wells were analyzed by the Quantichrom Calcium Assay Kit (Bio Assay Systems, Hayward, CA, USA), according to the manufacturer’s instructions.

#### 3.5.5. Statistics

Results are expressed as the mean ± SD. Statistical differences among the groups were analyzed by Tukey’s multiple comparison test (*p* < 0.05).

## 4. Conclusions

This study provides early evidence of the bioactive properties and biocompatibility of innovative porous glass coatings for potential use in hip joint prosthesis applications. The trabecular glass coatings, intended to be in contact with the host bone once implanted in vivo, exhibit a clear HA-forming ability in vitro, which can be considered as a precondition for bonding to bone in the body. No problems of interfacial delamination were assessed at the end of in vitro experiments. The good biological behavior of the coatings was assessed by the proliferation of mesenchymal and osteoblast-like cells, associated with the increasing synthesis of calcium nodules over time compared to uncoated ceramic substrates.

## Figures and Tables

**Table 1:** Composition of the starting glasses (moL.%).

Glass Code	SiO_2_	CaO	Na_2_O	Al_2_O_3_	P_2_O_5_	B_2_O_3_	Reagents
S57A7	57	30	6	7	-	-	SiO_2_, CaCO_3_, Na_2_CO_3_, Al_2_O_3_
S50B2	50	35	7	-	6	2	SiO_2_, CaCO_3_, Na_2_CO_3_, Ca_3_(PO_4_)_2_, H_3_BO_3_

## Abbreviations

BO	Bridging Oxygen
CI	Cell Index
CM	Confocal Microscopy
EDS	Energy Dispersive Spectroscopy
FBS	Fetal Bovine Serum
HA	Hydroxyapatite
MNC	Mononuclear Cells
MSC	Mesenchymal Stem
NBO	Non-Bridging Oxygen
PVA	Polyvinyl Alcohol
SBF	Simulated Body Fluid
SEM	Scanning Electron Microscopy
TEM	Transmission Electron Microscopy
